# Obesity-Related Factors in Adult Women with Early Menarche

**DOI:** 10.3390/healthcare11040557

**Published:** 2023-02-13

**Authors:** Hunha Cho, Jeong-Won Han

**Affiliations:** 1 College of Nursing, Kosin University, Busan 49104, Republic of Korea; 2 College of Nursing Science, Kyung Hee University, Seoul 02447, Republic of Korea

**Keywords:** obesity, menarche, women

## Abstract

The average menarche age among South Korean women has decreased over time. Early menarche in women causes a higher incidence of obesity owing to the continuous fat accumulation induced by prolonged exposure to estrogen and adrenal steroids. Identifying the factors affecting obesity in women who experience early menarche is essential for managing obesity in adult women. This study aimed to analyze the factors associated with obesity in adult women who experienced early menarche and to provide basic data for obesity management. This study was a cross-sectional and descriptive survey from the seventh Korea National Health and Nutrition Examination. A total of 371 women aged ≥ 19 years experienced early menarche, and the propensity matching method was used to analyze the obesity-related factors identified in previous studies. The results showed that obesity in adult women with early menarche was negatively associated with the level of aerobic exercise (OR = 0.53, 95% CI = 0.30–0.93, *p* = 0.028) and muscle-strengthening exercise (OR = 0.33, 95% CI = 0.17–0.64, *p* = 0.001). Future longitudinal studies on girls who experience early menarche are needed to address female obesity prevention at every stage of life, and, based on these findings, obesity management programs can be developed and applied and their effectiveness determined.

## 1. Introduction

The World Health Organization [1] classifies obesity as a disease and simultaneously presents it as the main contributing factor for colon, endometrial, ovarian, prostate, renal, breast, liver, and gallbladder cancers. Obesity causes metabolic diseases, such as diabetes, hypertension, hyperlipidemia, coronary artery disease, and metabolic syndrome. It also causes diseases of the reproductive and endocrine systems, such as female sexual precocity, irregular menstruation, and polycystic ovary syndrome [2]. In South Korea, the medical and caregiver costs incurred owing to obesity increased from USD 4.1 million in 2006 to USD 9.1 million in 2015, showing a consistent increase in socioeconomic losses [3]. Accordingly, the South Korean government considers obesity a disease that needs to be managed at the national level and attempts to prevent obesity through active policy interventions [4]. 

The obesity rate in South Korea increased from 31.8%, 37.7%, and 25.1% among adults, men, and women in 2013, to 34.6%, 42.8%, and 25.5% in 2018, respectively [2]. Although obesity is relatively less common among women than among men in South Korea, female obesity tends to increase with age because women easily accumulate fat. This is due to decreased basal metabolism and exercise, and because the muscle mass decreases while the fatty tissue increases, owing to a reduction in female hormone levels after menopause [5]. Furthermore, a longitudinal survey of Finnish women aged >31 years showed that women who experienced early menarche had a relatively high body mass index (BMI) after reaching adulthood. This indicates that obesity in women is related to early menarche [6]. Early menarche in women is reported to cause a higher incidence of obesity, owing to the continuous maintenance of fat accumulation by prolonged exposure to estrogen [7] and adrenal steroids [8]. Early menarche refers to menstruation beginning at an early age; despite slight differences among studies, it generally means that the onset of menstruation occurs at the age of 11 years or while in the 4th grade [9,10]. The average age of menarche onset among South Korean women has decreased over time. Kang and Oh [11] identified early sexual maturation, with the average menarche age as 12.3 years in girls born in the 1980s and the 1990s; however, it was 12.0 years in 2019. This suggests a possible rising trend in female obesity and encourages healthcare providers to focus on the girls’ menarche age to prevent female obesity and its associated complications. In particular, health care providers should consider active management from the time of early menarche in young girls to prevent adult female obesity. 

The identification of obesity-related factors is crucial to prevent and manage obesity in adult women who experience early menarche. In particular, South Korean women showed significant differences in body weight according to their educational level; specifically, the association index between women with low educational levels and being overweight increased from 5.0 in 2010 to 6.3 in 2014, which was significantly higher than the association index of 1.3% in the United States and the highest among the eight countries surveyed [12]. Obesity among adult women in Zimbabwe, a developing country, is seen to increase with increasing age and number of births, higher socioeconomic status, being married, and being employed [13]. However, the risk of obesity is high in developed Western countries [14]. Obesity is also related to lifestyle factors, such as sleep duration, sitting time, smoking and drinking, dietary habits, and physical activity. Sitting time, such as watching TV for a prolonged period, is a risk factor for obesity because it decreases physical activity, resulting in reduced energy consumption by the body [15]. Regular walking reduces the risk of obesity [16], whereas the risk increases with <6 h of sleep per day [17], drinking [18], fast food intake [19], and smoking [20]. In particular, smoking was more closely related to obesity in women than in men and was found to have the most significant influence on abdominal obesity when age, BMI, alcohol consumption, and physical activity were controlled [18,20]. Obesity has also been associated with stress, and it can be seen that the lower the stress in women, the lower the risk of obesity [16]. Low-income households have relatively limited choices for healthy food and weight-management activities [21]. The children living in low-income households have a relatively low intake of fresh seasonal fruits and vegetables and a high intake of food [22], In relation to physical activity, the frequency of moderate-intensity physical activity was low and sedentary time was high, resulting in an increase in BMI [23].

Obesity in adult women who experienced early menarche is associated with various complex factors [6]. Obesity eventually causes many problems, including an increase in obesity-related medical expenses, familial burden, social costs, various adult diseases and cancers, and a decrease in the quality of life. Accordingly, identifying obesity-related factors in adult women who experienced early menarche is important from both personal and social perspectives. Research predicting obesity in adulthood based on childhood health status is limited; furthermore, most previous studies that explained adulthood obesity did not differentiate between the sexes. In particular, research on women who experienced early menarche is lacking. In this respect, identifying the predictors of obesity in women who experienced early menarche is considered significant for the early management of obesity in adult women. Additionally, among the various methods of confirming the relevant risk factors for obesity, propensity score matching is a quasi-experimental design that cannot randomly assign study participants to experimental and control groups for comparison. One of the methods for estimating the relationship in a situation is to calculate the propensity score and perform a pairing [24]. By solving the problem of selection bias between the two groups through pairing, it is possible to estimate the treatment effects plausibly, and the propensity score method is also used in research studies other than experimental studies to identify factors related to a specific problem among patients [25]. Therefore, this study aimed to analyze obesity-associated factors in adult women who experienced early menarche. We also investigated the health-related habits associated with obesity.

## 2. Materials and Methods 

### 2.1. Participants and Sampling

The Korea National Health and Nutrition Examination Survey (KNHANES) [26] studies about 10,000 household members who are aged ≥1 y by probability sampling of 25 households in 192 areas of the country each year. As the KNHANES was reorganized into an annual survey system from 2007, a rolling sampling design was applied so that each year could be a nationally representative probability sample with independent cyclic samples. The survey participants were divided into young children (1–11 years), adolescents (12–18 years), and adults (19 years or older) according to their life cycle characteristics, and appropriate survey items for their characteristics were used. The participants of the present study were adult women aged ≥19 years who participated in the seventh (third year, 2018) KNHANES. The specific selection criteria were as follows: the participants had not attained menopause, had experienced menarche before 11 years of age, were still menstruating, had no restrictions on their physical activity due to physical or mental diseases, were not currently pregnant, and did not have mental illnesses or communication problems during the survey. The minimum number of participants required for the logistic regression analysis employed in the present study was 206, with an odds ratio (OR) = 1.7, α = 0.05, and power = 0.95, which was calculated using G*Power 3.1.3, based on a previous study [27,28] on obesity among South Korean women and the method presented by Hsieh, Bloch, and Larsen [27]. Thus, 371 participants were recruited for the present study and deemed appropriate for the logistic regression analysis.

### 2.2. Measurement 

The measurement used items suitable for the study purpose and were selected from those used in the National Health and Nutrition Examination Survey. Public health experts developed the items used in the National Health and Nutrition Examination Survey.

#### 2.2.1. Obesity

Body mass index was used to determine obesity and following the suggestion of the Korean Society for the Study of Obesity [2], participants were classified into normal weight, underweight, and obese groups for BMI 18.5–24.9 kg/m^2^, <18.5 kg/m^2^, and 25 kg/m^2^, respectively. Additionally, the participants were classified into obese and non-obese (healthy and underweight, respectively) groups. The Korean Clinical Guidelines Committee evaluated that a BMI ≥25 kg/m^2^ was reasonable as an obesity criterion for preventing the risk of chronic diseases. Unlike Korea, Western countries such as the United States define obesity as a BMI of 30 kg/m^2^ or more, considering the risk of death; therefore, the domestic standard is stricter.

#### 2.2.2. Propensity Score Matching Variables

##### Age

An open-ended question, “How old are you?”, was used to determine the age of the participants. According to the results of the National Health and Nutrition Examination Survey [26], the obesity rate of women in Korea is continuously increasing from the age of 30 years or older, and we reclassified the participants into those aged <30 and ≥30 years.

##### Education Level

The original data classified the educational level as elementary school or lower, middle school graduate, high school graduate, and college graduate or higher. The education levels were reclassified as lower than high school graduates, college graduates, and more senior.

##### Marital Status

The original data, which classified marital status into married and unmarried, were used.

##### Employment Status

The occupation reclassification and unemployment/non-economic activity status were used. The original data were used for the classification of occupation, which included managers, experts, related workers, office workers, service and sales workers, and skilled agriculture, forestry, and fishery workers. Furthermore, there were skilled workers, machine operators, assembly workers, simple laborers, and unemployed workers (e.g., homemakers and students). In the present study, the participants who were classified into different occupations and unemployed were reclassified into the employed and unemployed groups, respectively.

##### Personal Income

Personal income was based on the equal personal income surveyed by the KNHANES team and was divided into low, middle, and high incomes. The equalized personal income was the individual income obtained by converting the household income into each household member’s income.

##### Chronic Disease

The participants were determined to have a chronic illness if they were diagnosed with chronic diseases, such as hypertension, hyperlipidemia, stroke, angina, myocardial infarction, diabetes, thyroid disease, renal insufficiency, osteoarthrosis, rheumatoid arthritis, osteoporosis, pulmonary tuberculosis, asthma, stomach cancer, liver cancer, colon cancer, breast cancer, cervical cancer, lung cancer, other cancers, or hepatocirrhosis. The participants were reclassified as not having a chronic disease only if they did not have any one of these diseases.

##### Experience of Childbirth and Breastfeeding

One datum each was used for childbirth and breastfeeding. These were in the form of the following questions: “Have you ever given birth” and “Have you experienced breastfeeding after giving birth?”, respectively.

##### Whether Taking Oral Contraceptives

One datum was used to determine whether the individual was taking oral contraceptives.

#### 2.2.3. Variables Related to Obesity

##### Stress Recognition Rate

For stress recognition, the responses of the subjects to the question “How much do you feel stressed in daily life?” were reclassified (“very much” and “very much” into “high,” “little bit” and “seldom” into “low”).

##### Sleeping Time

The sleep duration on weekdays included the average hours of sleep per day, and that on weekends was calculated based on the participants’ bedtime and wake-up time. Additionally, they were based on a report by the Korean Society for the Study of Obesity (2014), which stated that ≥7 h of sleep was related to obesity. The sleep duration in the present study was divided into ≥8 h, 7–8 h, and <7 h per day.

##### Monthly Drinking Rate

The monthly drinking rate was calculated as a fraction of the number of months in which the participant drank less than once during the past 1 year, and then the participants were grouped into “less than one drink” and “one drink or more” per month groups.

##### Current Smoking

The proportion of current smoking was calculated as the current smoking rate to the total lifetime smoking of five packs (100 cigarettes) or more, and the participants were classified into past smokers, non-smokers, and current smokers. The past smokers and non-smokers were reclassified into “No”, and the current smokers were reclassified into “Yes”.

##### Muscle Strength Exercise (per Week)

The days of strength training were the number of days the participants performed resistance training, including push-ups, sit-ups, dumbbells, barbells, and pull-up bars, during the previous week. The present study divided the participants into a group that performed such activities once or more during the previous week and a group that did not.

##### Aerobic Exercise Rate (per Week)

The rate of aerobic exercise was calculated as a fraction of the time that the participants practiced physical activities at a moderate intensity for 2 h or more per week, high intensity for 1 h and 15 min or more, or a mix of medium and high intensity (1 min high intensity and 2 min low intensity), for the time corresponding to each activity. The participants were further classified into a group that performed aerobic exercise and a group that did not.

##### Nutrition State

The KNHANES provides raw data for calculating the total energy, carbohydrates, and fats based on the frequency of dietary intake in the past year. The Korean Diabetes Association [29] recommends that adults consume <50–60% of their total energy intake as carbohydrates and 25% as fats. Accordingly, in this study, the participants who consumed more than the KNHANES-recommended amount of total energy, in terms of carbohydrates and fats, were classified into the high-carbohydrate and high-fat diet groups, respectively.

### 2.3. Data Collection and Analysis

The data were obtained from the KNHANES website (https://knhanes.cdc.go.kr/knhanes/main.do (accessed on 3 November 2022)). This study analyzed the data obtained using SPSS 25.0 (Data solution. Inc., Seoul. Korea) and R 3.3.3 version (https://www.r-project.org/ (accessed on 3 November 2022)). Frequency, percentage, mean, and standard deviation have been used to describe the participants’ general characteristics. This study also utilized the propensity matching method by setting the age, education level, marital status, occupational status, household income, personal income, chronic disease status, childbirth status, breastfeeding status, and contraceptive use status as the matching variables. The nearest method was used as the matching algorithm, and the discard and caliper were both set to 0.25. Changes in the matching variables of the two matched groups were confirmed using standardized mean differences. A chi-square test was performed to understand the differences in the characteristics of the two groups selected using the propensity matching method. Logistic regression analysis was performed to identify the obesity-related factors in the participants. The logistic regression model calculates the likelihood ratio to test the significance of the model and estimates the parameters (logistic regression coefficients) using the maximum likelihood method. Likelihood refers to the probability of the occurrence of an observation under the given parameter estimates. The −2 log-likelihood, an index obtained by multiplying the logarithm of this value by −2, was used as a measure of how well the estimated model fit the data. Moreover, this study tested the goodness-of-fit of the Hosmer–Lemeshow test model and checked the degree of agreement between the actual value of the dependent variable and the value predicted by the model using the chi-square value.

## 3. Results

### 3.1. Participants’ Characteristics

A total of 193 (52.3%) participants were 30 years or older, 178 (47.7%) were younger than 30 years, 220 (60.4%) completed college or higher, 151 (39.6%) had graduated from high school, 189 (51.1%) were single, 182 (51.1%) were married, 222 (61.0%) were unemployed, 149 (39.0%) were employed, and most had a medium-level personal income. Moreover, 253 (67.4%) participants had chronic disease, 128 (32.6%) had no chronic disease, 211 (57.7%) had no childbirth experience, 235 (65.0%) had no breastfeeding experience, and 315 (89.1%) did not take oral contraceptives.

### 3.2. Participants’ Propensity Score Matching and Characteristic Comparison

**Figure 1:**
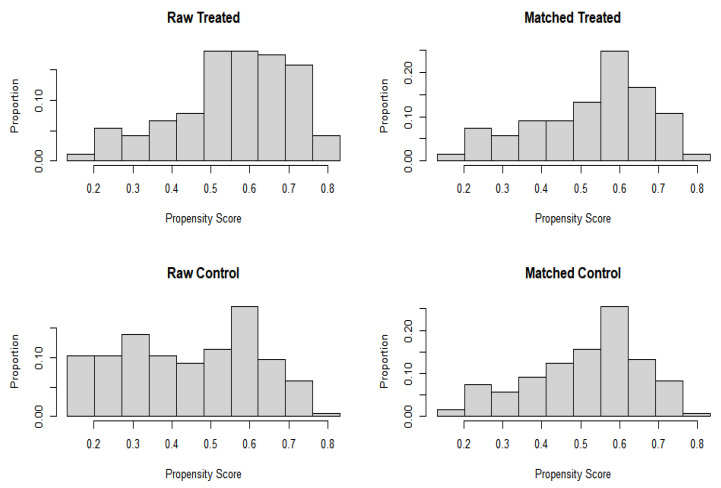
Data balance after propensity score matching.

**Figure 2:**
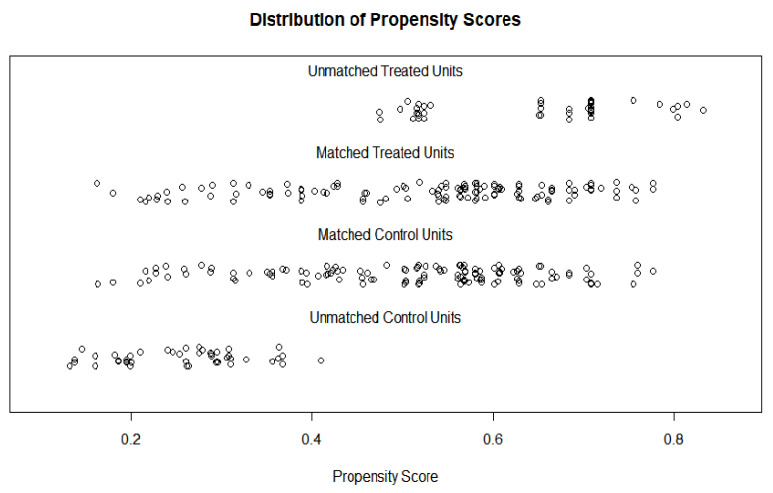
Distribution after propensity score.

Among the 371 participants, 240 (121 in the obese group and 119 in the non-obese group) were selected using the propensity matching method (Table 1; Figure 1 and Figure 2). Furthermore, the results showed that none of the matching variables were significantly different between obese and non-obese women after the propensity score matching (Table 2).

### 3.3. Obesity-Associated Factors in Adult Women Who Experienced Early Menarche

A logistic regression analysis was performed to identify the obesity-related factors in adult women who experienced early menarche. The results showed that the −2 LL and chi-square values (χ2 = 4.03, *p* = 0.038), which were used to test the fitness of the model, were statistically significant. The results confirmed that the aerobic exercise practice rate of participants (OR = 0.53, 95% confidence interval [CI] = 0.30–0.93, *p* = 0.028) and days of strength training (OR = 0.33, 95% CI = 0.17–0.64, *p* = 0.001) were the obesity-related factors (Table 3).

## 4. Discussion

This study aimed to provide baseline data for obesity management by analyzing the obesity-related factors in adult women who experienced early menarche. Early menarche occurs because the secretion of estrogen and gonadotropin-releasing hormone is activated at an early age. Moreover, the concentration of vitamin D in the blood affects the estrogen level [30]. Previously, Kim [31] reported that early menarche was related to obesity. This study also confirmed that 13% (371 people) of the 2849 female participants in the KNHANES experienced early menarche. After propensity score matching, 120 participants were confirmed as obese. This phenomenon is commonly found in various countries, such as across Europe, the United States, South Korea, and Saudi Arabia. A study on Danish female twins who experienced early menarche found that they had a higher risk of asthma [32]. Participants who experienced menarche before 12 years of age showed a higher incidence of metabolic syndrome and cardiovascular mortality rate [33]. Since early menarche is known to be associated with the risk of disease, it is necessary to continually manage the health of women who experienced early menarche right from childhood or adolescence instead of at adulthood and continue to manage their health for a long time. It is also necessary to develop and support various programs to prevent obesity from becoming a social health issue in adulthood by involving parents and students at home, school, and in society.

The results of this study confirmed that the rate of aerobic exercise practice and the number of days of strength training were the obesity-related factors in adult women who experienced premature menstruation. Recent obesity management guidelines emphasized the importance of the type and amount of exercise (a function of weekly frequency and exercise time), rather than the exercise intensity [34]. In particular, 300–420  min of moderate-intensity aerobic exercise per week is recommended [35]. Moreover, the amount of aerobic exercise should be increased weekly to significantly reduce weight in adults, and aerobic exercise is emphasized as a non-pharmacological therapy, especially for exercise related to menstruation [36]. Aerobic exercise improves cardiorespiratory function by supplying oxygen and blood to the muscles required for performing activities. Because aerobic exercise promotes energy metabolism using fat, it is essential to prevent obesity-related diseases by suppressing adipose tissue accumulation due to obesity [37]. The results of this study confirmed the effectiveness of aerobic exercise in controlling obesity by reducing stress, promoting lipid metabolism, and lowering leptin concentrations. Therefore, medical practitioners may recommend aerobic exercise programs for women who have experienced premature menstruation. Moreover, it is necessary to help women actively manage their health through the activation and publicity of community programs.

Additionally, Blakeley et al. [38] reported that strength training, identified in this study as an obesity-related factor, was effective at reducing obesity and improving lipid metabolism. Costa et al. [39] revealed the association of strength training with the maturation of growing children. The participants of this study were women who had experienced premature menstruation. Moreover, it could not be confirmed whether strength training had been practiced continually since the growth period, which is a limitation of this study. Since the participants who experience premature menstruation are known to have a high risk of obesity, it would be effective to apply a complex exercise program, including strength training and aerobic exercise, in such adults. Because several exercises have been introduced recently, it is important for patients with obesity to utilize an exercise program that suits their body and health conditions, which would require the cooperation of medical personnel and exercise experts. It is also necessary to prepare a human resource and system that can professionally manage people who have experienced premature menstruation over the long term.

## 5. Conclusions

This study aimed to provide baseline data to clinical practitioners to manage obesity in women by analyzing obesity-related factors in adult women who experienced early menarche. The results showed that the rate of aerobic exercise practice and the number of days of strength training were the obesity-related factors in adult women who experienced early menarche. This study is important because it included participants who experienced early menarche in childhood and provided baseline data for managing the health of participants who had experienced early menarche to medical personnel in clinical practice.

## 6. Limitations of the Study

Although the results of this study are meaningful in that they confirmed obesity-related factors in adult women who experienced early menarche, this study was limited in identifying the related factors by tracking various obesity-related factors that have occurred since childhood. Therefore, future studies are needed to longitudinally track and confirm the factors that may cause obesity in adulthood in children who have experienced early menarche. In particular, since this study had a limitation in that it was not possible to more accurately confirm the period and the amount of physical activity of the participants, it is necessary to consider this in future studies.

## Figures and Tables

**Table 1:** Data balance after propensity score matching.

Variables	Before Propensity Score Matching			After Propensity Score Matching		
	Mean of Obese Group (n = 165)	Mean of Non-Obese Group (n = 166)	Standardized Mean Difference	Mean of Obese Group (n = 121)	Mean of Non-Obese Group (n = 119)	Standardized Mean Difference
Age	1.68	1.78	0.76	1.69	1.69	0.76
Education level	1.67	1.55	0.50	1.62	1.60	0.49
Marital status	1.59	1.43	0.50	1.54	1.50	0.50
Occupation	1.39	1,39	0.49	1,45	1,38	0.48
Personal income	2.54	2.34	1.16	2.46	2.44	1.18
Family income	2.96	2.79	0.99	2.90	2.90	0.98
Chronic disease	0.20	0.45	0.50	0.26	0.28	0.45
Labor delivery	1.64	1.51	0.50	1.60	1.58	0.49
Breast feeding	1.70	1.60	0.49	1.67	1.67	0.47
Contraceptive pill	1.91	1.87	0.33	1.88	1.89	0.31

**Table 2:** Homogeneity of subjects’ characteristics after matching.

Variables	Category	Obese Group (n = 121)		Non-Obese Group (n = 119)		χ^2^ (*p*)
		n	%	n	%	
Age (years)	Under 30	59	24.6	62	25.8	0.83 (0.660)
	30–39	39	16.3	32	13.3	
	Over 39	23	9.6	25	10.4	
Education level	Under middle school	50	20.8	44	18.3	0.48 (0.490)
	Middle school or more	71	29.6	75	31.3	
Marital status	Yes	59	24.6	55	22.9	0.16 (0.693)
	None	62	25.8	64	26.7	
Occupation	Yes	72	30.0	66	27.5	0.48 (0.490)
	None	49	20.4	53	22.1	
Personal income	Low	34	14.2	31	12.9	0.31 (0.958)
	Middle	28	11.7	31	12.9	
	High	26	10.8	25	10.4	
Family income	Low	10	4.2	11	4.6	0.38 (0.278)
	Middle-low	35	14.6	25	10.4	
	Middle-high	33	13.8	45	18.8	
	High	43	17.9	38	15.8	
Chronic disease	Yes	88	36.7	88	36.7	0.05 (0.830)
	None	33	13.8	31	12.9	
Labor delivery	Yes	49	20.4	47	19.6	0.03 (0.874)
	None	72	30.0	72	30.0	
Breast feeding	Yes	40	16.7	39	16.3	0.00 (0.963)
	None	81	33.8	80	33.3	
Contraceptive pill	Yes	15	6.3	13	5.4	0.13 (0.722)
	None	106	44.2	106	88.3	

**Table 3:** Obesity-associated factors in adult women who experienced early menarche.

Variables	B	SE	*p*	Exp (B)	95% CI of Exp (B)	
					Lower	Upper
Stress cognition rate	0.06	0.28	0.830	1.06	0.61	1.84
Sleeping time during week	0.15	0.28	0.591	1.16	0.67	2.02
Alcohol consumption rate	0.25	0.28	0.375	1.28	0.73	2.24
Smoking	−0.27	0.22	0.229	0.76	0.48	1.18
Aerobic exercise	−0.62	0.28	0.028	0.53	0.30	0.93
Muscle-strengthening exercise	−1.09	0.33	0.001	0.33	0.17	0.64
High-fat diet	−0.33	0.32	0.302	0.71	0.38	1.34
High-carbohydrate diet	0.13	0.35	0.695	1.14	0.57	2.28

SE = Standard error, 95% CI = 95% confidence interval.

## Data Availability

The datasets used and/or analysed during the current study available from the corresponding author on reasonable request.
